# Women and postfertilization effects of birth control: consistency of beliefs, intentions and reported use

**DOI:** 10.1186/1472-6874-5-11

**Published:** 2005-11-28

**Authors:** Huong M Dye, Joseph B Stanford, Stephen C Alder, Han S Kim, Patricia A Murphy

**Affiliations:** 1 Department of Family and Preventive Medicine, University of Utah School of Medicine, Salt Lake City, UT, USA; 2 College of Nursing, University of Utah School of Medicine, Salt Lake City, UT, USA

## Abstract

**Background:**

This study assesses the consistency of responses among women regarding their beliefs about the mechanisms of actions of birth control methods, beliefs about when human life begins, the intention to use or not use birth control methods that they believe may act after fertilization or implantation, and their reported use of specific methods.

**Methods:**

A questionnaire was administered in family practice and obstetrics and gynecology clinics in Salt Lake City, Utah, and Tulsa, Oklahoma. Participants included women ages 18–50 presenting for any reason and women under age 18 presenting for family planning or pregnancy care. Analyses were based on key questions addressing beliefs about whether specific birth control methods may act after fertilization, beliefs about when human life begins, intention to use a method that may act after fertilization, and reported use of specific methods. The questionnaire contained no information about the mechanism of action of any method of birth control. Responses were considered inconsistent if actual use contradicted intentions, if one intention contradicted another, or if intentions contradicted beliefs.

**Results:**

Of all respondents, 38% gave consistent responses about intention to not use or to stop use of any birth control method that acted after fertilization, while 4% gave inconsistent responses. The corresponding percentages for birth control methods that work after implantation were 64% consistent and 2% inconsistent. Of all respondents, 34% reported they believed that life begins at fertilization and would not use any birth control method that acts after fertilization (a consistent response), while 3% reported they believed that life begins at fertilization but would use a birth control method that acts after fertilization (inconsistent). For specific methods of birth control, less than 1% of women gave inconsistent responses. A majority of women (68% or greater) responded accurately about the mechanism of action of condoms, abstinence, sterilization, and abortion, but a substantial percentage of women (between 19% and 57%) were uncertain about the mechanisms of action of oral contraceptives, intrauterine devices (IUDs), Depo-Provera, or natural family planning.

**Conclusion:**

Women who believe that life begins at fertilization may not intend to use a birth control method that could have postfertilization effects. More research is needed to understand the relative importance of postfertilization effects for women in other populations, and in relation to other properties of and priorities for birth control methods. However, many women were uncertain about the mechanisms of action of specific methods. To respect the principles of informed consent, some women may need more education about what is known and not known about the mechanisms of action of birth control methods.

## Background

Many studies have investigated factors or barriers that are associated with women's use of birth control methods [1,2]. Using the theoretical framework of the Theory of Planned Behavior, some studies have investigated beliefs, attitudes, norms, and intentions that contribute to the use of specific contraceptive methods, either for contraception or the prevention of sexually transmitted infection [3-7]. One issue that has received little attention but that may be important for some women is understanding or beliefs and intentions about mechanisms of action of birth control methods.

While there is dispute among medical experts about whether pregnancy begins at fertilization or implantation [8-10], up to half of women in national polls in the United States believe that human life begins at fertilization [11]. For these women, a birth control method that could act occasionally after fertilization may conflict with personal, ethical or religious beliefs [12]. As a result, these women may wish to refrain from using birth control methods that may exhibit a postfertilization effect, even if the effect were to occur prior to the woman recognizing that she was pregnant. A recent study found that Latina women in the United States who believed that emergency contraception acted after fertilization were less likely to use it [13]. However, we are unaware of any study that has systematically investigated how women's beliefs about the beginning of human life and the mechanisms of action of birth control methods may relate to their intentions to use and actual use of specific birth control methods.

The primary purpose of our study was to determine among women beliefs about the mechanisms of action of birth control methods in relation to postfertilization effects and how this understanding related to their stated intentions to use methods of birth control, and reported use of specific methods. We aimed to do this by assessing among women: 1) beliefs about the mechanisms of action of select birth control methods, 2) personal belief of when human life begins, 3) intention to use birth control methods that may exhibit postfertilization effects, and 4) reported actual use of specific birth control methods. We assessed these by analyzing the consistency of responses to relevant questions of a questionnaire designed to address women's understanding and attitudes about the potential for postfertilization effects.

## Methods

### Questionnaire development

The questionnaire used was a 4-page, 37-item survey assessing the following in women: 1) past, present and future use of birth control methods, 2) personal decision to use a birth control method if it acted at a certain stage of the reproductive process (described further below), 3) personal belief about what stage(s) specific birth control methods may act, and 4) personal belief about when human life begins. An introduction to the early stages of human reproduction was provided within the questionnaire and briefly explained in three stages. These stages were explained as follows: "Stage 1 (Before fertilization – before the uniting of the sperm and the egg), Stage 2 (After fertilization but before implantation – after the egg is fertilized but before it implants in the uterus; usually this time period is 5–7 days), and Stage 3 (After implantation in the uterus)." Key questions were focused around these stages.

In constructing the questionnaire, we investigated all temporary methods of preventing unwanted birth (birth control), including abstinence, contraception, and abortion. For the purpose of assessing women's understanding of the early stages of reproduction described in the questionnaire, methods of birth control with unambiguous stages of action were designated in advance by the developers of the questionnaire. These were abstinence, condoms, sterilization, natural family planning (acting at Stage 1, prior to fertilization), and surgical or medical abortion (acting at Stage 3, after implantation). The questionnaire contained no information about how specific birth control methods work in relation to Stages 1, 2, or 3, but did contain questions asking women's own beliefs of how specific methods of birth control may act.

The questionnaire was compiled and assessed for face validity through the collaboration of experts in reproductive medicine, demography, and population issues. Pilot testing of the questionnaire was performed with 21 patients in Salt Lake City, Utah, and 15 patients and 10 nurses in Tulsa, Oklahoma. The pilot testing consisted of having the patients or nurses fill out the questionnaire in writing (as it was designed to be used), followed by an interview to discuss their understanding of the reproductive stages and concerns about the questionnaire and specific understanding of key questions. Those completing the pilot testing reported understanding of the reproductive stages and issues addressed by the questionnaire. As a result of pilot testing, wording was clarified in the questions about intentions to use birth control methods, questions were added to include women's understanding of Depo-Provera, and whether her partner had or planned to have a vasectomy. The questionnaire is available from the authors on request.

### Study population

The primary sites for this study included clinics in Salt Lake City, Utah and Tulsa, Oklahoma. In Salt Lake City, the sites included the Obstetrics and Gynecology clinic within the University of Utah Medical Center, a University of Utah family medicine clinic in the community, two private Obstetrics and Gynecology practices, and a Community Health Center. In Tulsa, Oklahoma, the site included was a teaching clinic associated with a family medicine residency program sponsored by a Protestant religious organization. None of the clinics in Utah were religiously affiliated or sponsored.

The study questionnaire was presented by research assistants (male medical students or female clinic staff) to English-speaking women between the ages of 18 and 50 while they waited for appointments during regular clinic hours in early 2002. The questionnaire was also presented to women below the age of 18 if the appointment involved obstetrical care or birth control counseling. The research assistant explained the study and asked whether the woman would like to participate by filling out the questionnaire. After each woman agreed or declined, she had no further interaction with the research assistant. The questionnaire was self-administered in writing and took approximately 10–15 minutes to complete. The questionnaire contained a statement that filling out the questionnaire constituted voluntary participation in the study and did not affect clinical care provided. No identifying information was collected on participants in the study. The study was approved by the University of Utah Institutional Review Board (IRB), which did not require written consent of patients, or of parents for patients under 18, since these patients were presenting for clinical care that did not require parental consent.

### Statistical analyses

Statistical analyses consisted of frequencies and cross-tabulations among the key questions. The intent was to determine the understanding, beliefs, and intentions of women concerning the postfertilization effects of specific methods of birth control in relation to their understanding and concern for postfertilization effects. Except when otherwise specified, all percentages reported in this paper are based on the denominator of all women included in the study.

## Results

### Demographics

A total of 928 self-administered questionnaires were distributed at participating clinics in the study. Of these 928, a total of 748 were filled out and returned, thus resulting in an 81% response rate. Of these 748, a total of 130 women were removed from analyses because they were over the age of 50, or had a condition or surgery (menopause, hysterectomy, tubal ligation, etc.) that made them unable to become pregnant for the rest of their lives. This resulted in 618 eligible participants that were included in the study.

About half (54%) of the participants came from clinics in Salt Lake City, UT, while the other half (46%) came from Tulsa, Oklahoma. The mean age of the study participants was 29 years. Most of the study participants were educated: 78% had a college degree or some college education, and 15% had a high school diploma. The majority (75%) were White, 4% were Black, 6% Hispanic, and 3% each were American Indian and Asian. A majority of the study participants were married (58%) and had a previous pregnancy (70%) or were currently pregnant (29%). A majority were religiously affiliated (71%), attended church at least once a week (50%), and many responded that their faith was a very important influence in their life (40%). The most commonly used methods of birth control by study participants were condoms and oral contraceptives (Table 1).

**Table 1:** Current Use of Selected Birth Control Methods (n = 618)

**Birth Control Methods**	**Percentage Using Now (or Near Future if Pregnant) (a)**
Condom	33.0 *(29.3,36.7)*
Vasectomy	9.4 (*7.1,11.7)*
Oral contraceptives	38.0 (*36.7,44.5)*
Intrauterine device	3.6 *(2.1,5.1)*
Depo-Provera	6.1 *(4.2,8.0)*
Withdrawal	7.9 *(5.8,10.0)*
Spermicide	2.4 *(1.2,3.6)*

(a)Parentheses include 95% confidence interval limits.

To assess the understanding of the mechanisms of action of birth control methods, women were asked to give their beliefs regarding at which stage(s) of human reproduction various methods of birth control may act (Table 2). In their responses to this question, women were reminded that some methods may act at more than one stage and encouraged to check all stages at which each method acts. A majority of women provided the designated single correct response for the following methods: 71% responded that abstinence acts at Stage 1, 84% responded that condoms act only at Stage 1, 68% responded that sterilization acts only at Stage 1, and 69% responded that abortion acts only at Stage 3 (note that percentages in Table 2 are higher because of inclusion of women that responded for more than one stage). There was less familiarity with medical abortion (RU-486), with only 39% of women responding that it acts at Stage 3, and natural family planning, with only 50% of women responding that it acts at Stage 1. With regard to beliefs about methods of birth control that were not designated in advance to have a single correct answer for mechanisms of action, a majority of women (70%) thought oral contraceptives act at Stage 1, but some (22%) also responded they act at Stage 2. Over half of women (56%) responded that emergency contraception acts at Stage 2. Other birth control methods that had high percentages that were marked "Unsure" by many women as to their mechanisms of action included progestin-only pills (57%), intrauterine devices (41%), and Depo-Provera (35%).

**Table 2:** Personal Belief about Stages at which Birth Control Methods May Act (n = 618)

**Birth Control Methods**	Percentage Responding (a)			
	
	**Stage 1 (b)**	**Stage 2 (b)**	**Stage 3 (b)**	**Unsure**
Abstinence	82.0 *(79.0,85.0)*	11.2 *(8.7,13.7)*	5.5 *(3.7,7.3)*	9.1 *(6.8,11.4)*
Condoms	86.3 *(83.6,89.0)*	2.8 *(1.5,4.1)*	1.9 *(0.8,3.0)*	7.8 *(5.7,9.9)*
Sterilization	75.6 *(77.2,79.0)*	7.8 *(5.7,9.9)*	5.5 *(3.7,7.3)*	15.7 *(12.8,18.6)*
Abortion	6.2 *(4.3, 8.1)*	14.7 *(11.9,17.5)*	83.7 *(80.8,86.7)*	8.4 *(6.2,10.6)*
RU-486	4.7 *(3.0,6.4)*	20.4 *(17.2,23.6)*	54.5 *(50.6,58.4)*	33.3 *(29.6,37.0)*
Oral Contraceptives	70.2 *(66.6,73.8)*	22.3 *(19.0,25.6)*	3.6 *(2.1,5.1)*	18.5 *(15.4,21.6)*
Progestin-only pills	27.4 *(23.9,30.9)*	13.9 *(11.2,16.6)*	1.8 *(0.8,2.8)*	57.3 *(53.4,61.2)*
Intrauterine Devices	39.5 *(35.6–43.4)*	20.2 *(17.0–23.4)*	6.0 *(4.1–17.9)*	41.1 *(37.2–45.0)*
Depo-Provera	54.9 *(51.0,58.8)*	9.1 *(6.8,11.4)*	2.4 *(1.2, 3.6)*	35.1 *(31.3,38.9)*
Emergency Contraception	11.8 *(9.3,14.3)*	56.6 *(52.7,60.5)*	18.1 *(15.1,21.1)*	26.9 *(23.4,30.4)*
Natural Family Planning	59.1 *(55.2,63.0)*	8.3 *(6.1,10.5)*	7.8 *(5.7,9.9)*	30.9 *(27.3,34.5)*

(a) Percentages do not add to 100% due to the selection of more than one stage as a response, missing responses and rounding. Parentheses include 95% confidence interval limits.

(b) Stage 1 – Before fertilization – before the uniting of the sperm and the egg; Stage 2 – After fertilization but before implantation; Stage 3 – After implantation in the uterus.

Women were asked if they would consider using a birth control method that acts at Stage 2 or Stage 3. 53% responded "No" to using a birth control method that acts at Stage 2, and 74% responded "No" to using a birth control method that acts at Stage 3. To assess the underlying understanding of these questions about the stages of human reproduction, we analyzed the consistency of responses regarding intention to use a birth control method that acts at Stage 2 and Stage 3. If a woman answered that she would not use a birth control method that acts at Stage 2 of human reproduction, she would most likely not use a birth control method that acts at Stage 3. Half (51%) of all women responded consistently with "No" to using a birth control method acting at Stage 2 and "No" to using a birth control method acting at Stage 3. Less than 1% of women responded inconsistently with "No" to using a birth control method acting at Stage 2 and "Yes" to using a birth control method acting at Stage 3.

Further evidence suggesting that a postfertilization effect may affect women's intention to use a birth control method is reflected in responses regarding intention to stop the use of birth control methods. Women were asked how their decision to use a birth control method would be affected if they were using a birth control method and learned that it acts at Stage 2 or Stage 3. 44% of women responded if there was even the remote possibility of the method acting at Stage 2, they would stop using it. 69% of women responded if there was even the remote possibility of the method acting at Stage 3, they would stop using it. If a woman answered she would not use a birth control method that acts at Stage 2 or Stage 3, then if she were to learn that she was using a birth control method that acts at Stage 2 or Stage 3 respectively, we expected that she would intend to stop using it. This would be a consistent response pattern. Of the entire group of respondents, 38% responded consistently that they would not use a method that acts at Stage 2 and they would stop using a method they were currently using if they learned it acts at Stage 2. An even higher percentage of respondents (64%) responded consistently that they would not use a method that acts at Stage 3 and that they would stop using a method they were currently using if they learned it acts at Stage 3.

If a woman, however, answered that she would not use a birth control method that acts at Stage 2 or Stage 3, then if she were to learn that she was using a birth control method that acts at Stage 2 or Stage 3 respectively, but still answered she would continue using it, we considered this to be an inconsistent response pattern. Another inconsistent response pattern would be if a woman answered that she would use a birth control method that acts at Stage 2 or Stage 3, then if she were to learn that she was using a birth control method that acts at Stage 2 or Stage 3 respectively, she would stop using it. Of the respondents, 4% responded inconsistently to questions pertaining to Stage 2, and 2% responded inconsistently to questions pertaining to Stage 3.

The personal belief of when human life begins for each woman is at the core of the question of using birth control methods that may act after fertilization. To assess belief of when human life begins, women were asked the open-ended question: "In your personal opinion, when does human life begin?" Most women reported the personal belief that human life begins at fertilization (48%). Some women had the belief that life begins after fertilization, but before implantation (5%), and some at implantation (5%) or later (14%). If a woman answered that human life begins at fertilization, she would most likely answer "No" to using a birth control method that acts at Stage 2 and at Stage 3. Of all the women in the study, 34% reported they believed that life begins at fertilization and would not use a birth control method that acts at Stage 2 nor use a method that acts at Stage 3, thus responding consistently. Conversely, 3% of all women responded to these questions inconsistently.

If a woman answered that human life begins at implantation, she would most likely answer "Yes" to using a birth control method that acts at Stage 2 but "No" to using a birth control method that acts at Stage 3. Of all the women in the study, 2% reported they believed that life begins at implantation and that they would use a birth control method that may act at Stage 2 but not at Stage 3, thus responding consistently. Inconsistent responses to these questions were given by 1% of all women in the study. These women reported they believed that life begins at implantation but would not use a birth control method that may act at Stage 2 or Stage 3.

Finally, we assessed the consistency of responses among women who stated the intention to not use any birth control method that acts at Stage 2 or Stage 3 with her personal belief of how specific birth control methods may act and her reported actual use of these same methods. Actual use was assessed in response to a question of which methods the woman was currently using or if she was pregnant, planning to use in the near future. In the Theory of Planned Behaviour (TPB), the most important determinant of behaviour is behavioural intention, which in turn, is affected by behavioural beliefs [14]. If a woman believed that a birth control method acts at Stage 2 or Stage 3 and did not intend to use a birth control method that acts at Stage 2 or Stage 3, she would most likely not be currently using or planning to use this method. This would be consistent. If a woman believed that a birth control method acts at Stage 2 or Stage 3 and answered "No" to using a birth control method that acted at Stage 2 or Stage 3, then if she was currently using or planning to use this method, this would be inconsistent with her beliefs and intentions. If a woman, however, did not believe that a birth control method acts at Stage 2 or Stage 3 and did not intend to use a birth control method that acted at Stage 2 or Stage 3, and then if she was currently using or planning to use this method, this would be consistent also. A higher percentage of women responded in this manner. The results to these analyses are given in Table 3, where the percentages reported are of all women analyzed in the study. The largest percentage of both inconsistent and consistent responses was reported for oral contraceptives pertaining to Stage 2. For oral contraceptives, 2% of respondents gave inconsistent responses while 24% of respondents gave consistent responses, 8% from women who were not using oral contraceptives and 16% from women who were using oral contraceptives.

**Table 3:** Consistency of Beliefs, Intention to Use and Actual Use of Birth Control Methods

	**Percentage Responding (a)**		
***Belief of how a specific method acts***	***Acts at Stage 2 *(b)**		***Does not act at Stage 2***

***Intention to use any method that acts at Stage 2 *(b)**	***No***		

***Actual use of the specific method ***	***No***	***Yes***	

**Response Consistency**	**Consistent (c)**	**Inconsistent (d)**	**Consistent (e)**

Condoms	1.0 *(0.2,1.8)*	0.8 *(0.1,1.5)*	14.6 *(11.8,17.4)*
Sterilization	4.1 *(2.5,5.7)*	0.3 *(0.0,0.7)*	4.7 *(3.0,6.4)*
Oral Contraceptives	8.3 *(6.1,10.5)*	1.5 *(0.5,2.5)*	16.2 *(13.3,19.1)*
Progestin-only pills	7.3 *(5.2,9.4)*	0.2 *(0.0,0.6)*	2.4 *(1.2,3.6)*
Intrauterine Devices	10.4 *(8.0,12.8)*	0.0 *(0.0,0.0)*	1.1 *(0.3,1.9)*
Depo-Provera	4.4 *(2.8,6.0)*	0.5 *(0.0,1.1)*	1.8 *(0.7,2.9)*

	**Percentage Responding (a)**		

***Belief of how a specific method acts***	***Acts at Stage 3 *(b)**		***Does not act at Stage 3***

***Intention to use any method that acts at Stage 3 *(b)**	***No***		

***Actual use of the specific method***	***No***	***Yes***	

**Response Consistency**	**Consistent (c)**	**Inconsistent (d)**	**Consistent (e)**

Condoms	0.5 *(0.0,1.1)*	0.8 *(0.1,1.5)*	23.0 *(19.7,26.3)*
Sterilization	4.5 *(2.9,6.1)*	0.2 *(0.0,0.6)*	6.8 *(4.8,8.8)*
Oral Contraceptives	1.9 *(0.8,3.0)*	0.5 *(0.0,1.1)*	27.4 *(23.9,30.9)*
Progestin-only pills	1.5 *(0.5,2.5)*	0.2 *(0.0,0.6)*	4.4 *(2.8,6.0)*
Intrauterine Devices	5.7 *(3.9,7.5)*	0.0 *(0.0,0.0)*	2.8 *(1.5,4.1)*
Depo-Provera	1.6 *(0.6,2.6)*	0.2 *(0.0,0.6)*	4.4 *(2.8,6.0)*

(a) Percentages are of all respondents to questionnaire who gave the combined responses indicated in the particular column for each specific birth control method. Parentheses include 95% confidence interval limits.

(b) Stage 2 – After fertilization but before implantation; Stage 3 – After implantation in the uterus.

(c) This column includes respondents who gave the following internally consistent responses: they would not use a method that acted at Stage 2 or 3, believed that the specific method acted at Stage 2 or 3, and reported no actual use of the method.

(d) This column includes respondents who gave the following internally inconsistent responses: they would not use a method that acted at Stage 2 or 3, believed that the specific method acted at Stage 2 or 3, and reported actual use of the method.

(e) This column includes respondents who gave the following internally consistent responses: they would not use a method that acted at Stage 2 or 3, believed that the specific method did not act at Stage 2 or 3, and reported actual use of the method.

## Discussion

These data demonstrate a strong consistency in the women studied for beliefs about postfertilization effects of specific birth control methods, beliefs about when human life begins, and the intention to use specific birth control methods. These findings are consistent with the Theory of Planned Behavior, which proposes a direct relationship between beliefs, intentions, and behavior [14], and specifically with previous studies of the Theory of Planned Behavior that have shown a consistency of beliefs, attitudes, intentions, norms, and behavior for other issues affecting contraceptive use, including overall attitudes about specific contraceptive methods, beliefs about the benefits, utility, and disadvantages of specific contraceptive methods, subjective norms about contraception, beliefs about self-control, and partners' attitudes [3-7,15].

In the Theory of Planned Behavior (TPB), an individual's behavioural beliefs and evaluations of behavioural outcomes are contributing factors that affect an individual's attitude toward the behaviours. This attitude toward the behaviour has an impact on behavioural intention, which in turn, affects behaviour. Applying the TPB, a women's personal beliefs concerning when human life begins may influence her intention to use birth control methods that may exhibit postfertilization effects, and therefore, her actual use or lack of use of methods that exhibit such effects. The strength of the association in our study suggests that for many of these women, the belief that human life begins at fertilization, the belief that a birth control method may sometimes work after fertilization or after implantation may be an important factor in intentions about which method of birth control to use, and in actual use. However, there are components of the Theory of Planned Behavior that our study did not directly address. These include attitudes, subjective norms, and self-efficacy.

Contraceptive decision making in women is a complex process. A recent study has suggested a model for contraceptive decision making in women that involves three steps that are consistent with and complementary to the Theory of Planned Behavior [16]. These three steps are becoming aware, navigating a course and weighing what is best personally for each individual. This could be applied to the specific issues of postfertilization effects addressed by our study. As women become aware of the possibility of postfertilization effects, their intention to use a birth control method that may exhibit these effects may decrease if this conflicts with their moral and ethical beliefs. Similarly, the Health Belief Model, which is closely related to the Theory of Planned Behaviour, can be applied to this situation [14]. Applying the Health Belief Model, women who determine that specific birth control methods may have postfertilization effects that are in conflict with their moral or ethical beliefs (perceived severity) and are using such methods (perceived susceptibility) may develop negative attitudes toward these methods and discontinue their use (self-efficacy).

In our questionnaire, the specific beliefs, intentions, and behaviors we measured were all single-item measures. Thus, this questionnaire was not a psychometric instrument, where different responses are presumed to be measuring the same underlying psychometric construct (such as stress or pain). Therefore standard approaches to assessing reliability (such as Cronbach's alpha) are not appropriate in this context. We also did not address the issues of test-retest reliability or response shift in this study. Future studies should attempt to elucidate more thoroughly the nature of the underlying beliefs, women's exact understanding of reproductive physiology, test-retest reliability, and response shift. In addition, future studies should examine other components of the Theory of Planned Behavior that we did not directly address: namely norms, attitudes, and self-efficacy.

As presented in Table 2, we have used the term "birth control" as an umbrella term for methods that are used to prevent unwanted birth, including abstinence, natural family planning, contraception, emergency contraception, and abortion. While some advocates of reproductive rights consider each of these options and services related to them as legitimate and essential means to prevent unwanted births [17-20], others would accept some but reject others as being included appropriately in the term "birth control." Our use of the term "birth control" in this study is not meant to discount differences between these methods or approaches, nor to imply that they are all equally acceptable to women. Our purpose in this study was rather to investigate how women understand the mechanisms of action of the various interventions that can be undertaken to prevent unwanted birth, and the influence of this understanding on intentions. Because we were interested in studying birth control intentions among women who actually may face those choices, we excluded women who had undergone tubal ligation, who had undergone menopause, or who were otherwise sterile.

The literature has focused on many properties considered by women when selecting a contraceptive method, such as whether a contraceptive method is highly effective, has a prolonged duration of action, is rapidly reversible, protects against sexually transmitted diseases, is easily accessible, is convenient/easy to use, and poses little to no side effects, risks, or disadvantages [21,22]. Some studies have considered that a disadvantage of a contraceptive method could be if it contradicted a woman's moral or religious beliefs, but have not specified such issues in more detail [23,24]. As noted, a recent study did find that many women were unwilling to use emergency contraception if they believed that its mechanism of action was after fertilization because this contradicted their moral beliefs [13]. In our study, this issue not only held true for emergency contraception, but for any birth control method that women believed had postfertilization effects as one of its mechanism of action. However, we did not study the relative weight that the potential for postfertilization effects may carry in relation to other contraceptive properties that have been studied more extensively. In our study there were few women who reported they did not intend to use a method with postfertilization effects, believed that a specific method had postfertilization effects, and still reported actual use of that specific method.

Since the potential for postfertilization effects is important to many women in this study, it is also remarkable that many of them were unsure about the mechanisms of action of specific methods of birth control. Patients today have emphasized a need for more information from physicians and healthcare providers [25,26]. Though information concerning birth control methods and the potential for postfertilization effects may be difficult to provide due to uncertainty about the exact mechanisms of action and the exact frequency of these mechanisms of action for many methods of birth control, an attempt to present what is known and not known about the mechanisms of action of specific methods may be important for adequate informed consent for some women [27-33]. Without reasonably complete information, women may be denied the opportunity to make a fully informed decision during contraceptive selection.

The present study has several limitations. The women included in the study are not a representative sample of all the women in the United States. A majority of women in our study were well educated and White. The religiosity of women in this particular clinic-based sample may not reflect that of the general United States population, because of the States in which the clinics are located (Utah and Oklahoma), and the religious affiliation of the clinic in Oklahoma.

We recognize the limitations of the questionnaire used in the study. The questionnaire was developed for face validity by consultation with experts in the field and was pilot-tested to determine if the issues addressed were understood by women but has not been validated against other measures because we are unaware of other measures that specifically address these questions. We also recognize the controversy in asking the open-ended question "When does human life begin?" since this is a question that involves moral and religious values and a personal perception of life itself. Nevertheless, our research supports the proposal that beliefs about the beginning of human life are highly relevant to some women and their reproductive choices. In the pilot testing of the questionnaire, we found that women understood the early stages of human reproduction as presented, but we did not directly assess women's understanding of these issues in the main study sample. Without a prior knowledge of the stages of human reproduction, it may be a challenging task to assimilate and absorb new information and then attempt to apply it during the short time period of filling out the questionnaire. Patients may not have previously considered these issues prior to receiving the questionnaire. Some of them may have needed additional time to contemplate such sensitive issues prior to answering the questions that address such topics. Because of these issues, the rate of unsure and inconsistency of responses may be inflated in these data. Despite this limitation, the level of consistency in key responses within this questionnaire was high.

## Conclusion

The strong consistency of responses for beliefs, intentions, and actual use of birth control methods in this study indicate that the potential for postfertilization effects may be important in some women's intention to use and actual use of specific birth control methods. Further research is needed to understand the relative importance of this issue in different populations and in relation to other priorities and properties of birth control methods. In the meantime, we believe that it is important that physicians and health care providers who prescribe birth control methods be willing to educate women on the basic processes of human reproduction and what is known about the mechanisms of action of birth control methods. For women who believe that life begins at fertilization, this education may be especially important since a birth control method that can exhibit a secondary postfertilization effect may conflict with their ethical or religious beliefs. This information may be necessary for some patients to make a fully informed decision during the process of birth control method selection.

## Competing interests

The author(s) declare that they have no competing interests.

## Authors' contributions

Huong M Dye designed and performed the analysis and wrote the manuscript. Joseph B Stanford conceived the study, supervised the analysis and revised the manuscript. Stephen C Alder, Han S Kim, and Patricia A Murphy provided additional guidance in formulating the analysis and revising the manuscript.

## Pre-publication history

The pre-publication history for this paper can be accessed here:


